# Supplementary figures and images for: Metabolic, Organoleptic and Transcriptomic Impact of Saccharomyces cerevisiae Genes Involved in the Biosynthesis of Linear and Substituted Esters
Source: Int J Mol Sci. 2021 Apr 14;22(8):4026. doi: 10.3390/ijms22084026 (PMC8070738; doi:10.3390/ijms22084026)

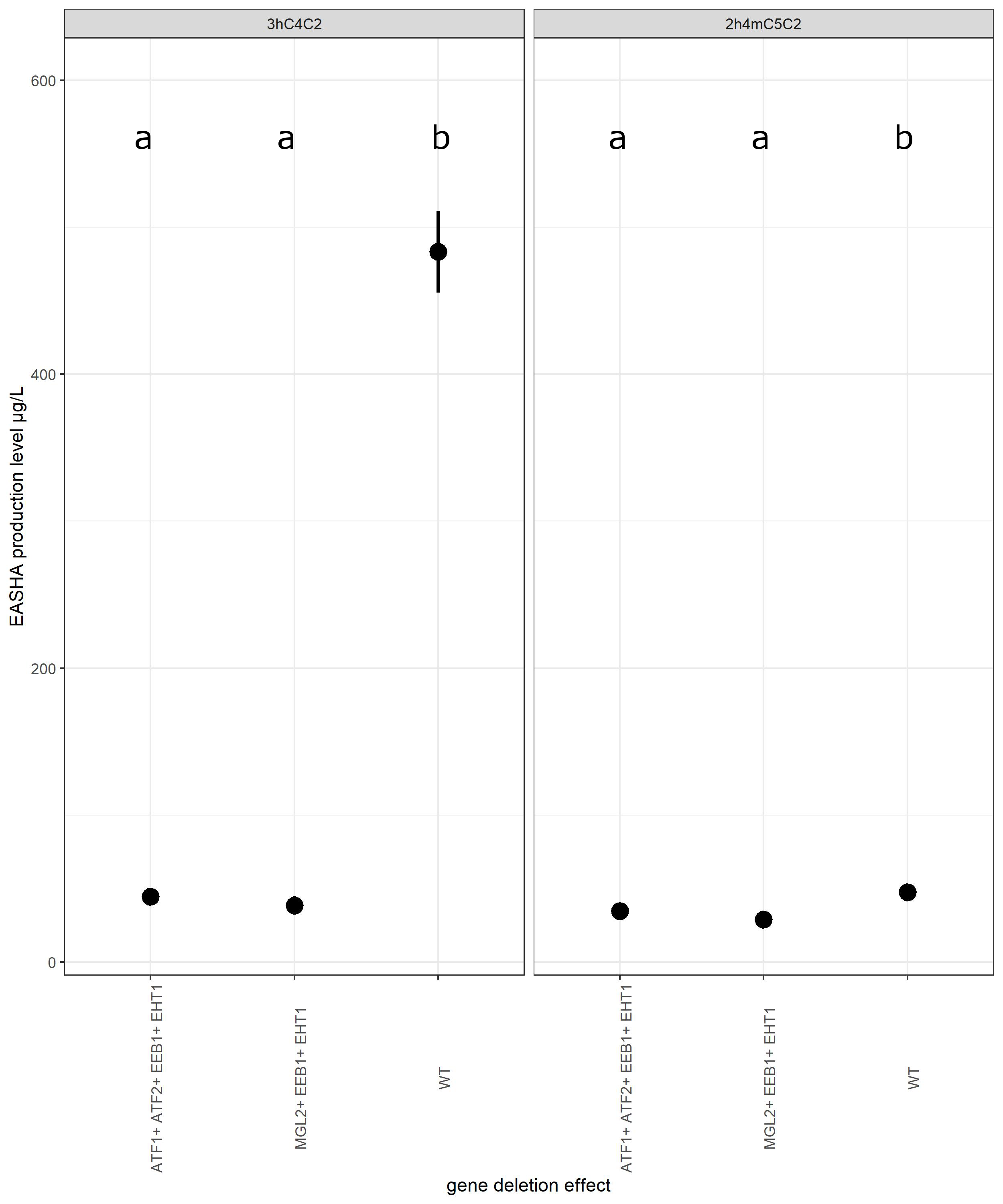

Supplement: Supplementary file 1 [file ijms-22-04026-s001.zip › TABLES et STABLE/Figure S1.tif]

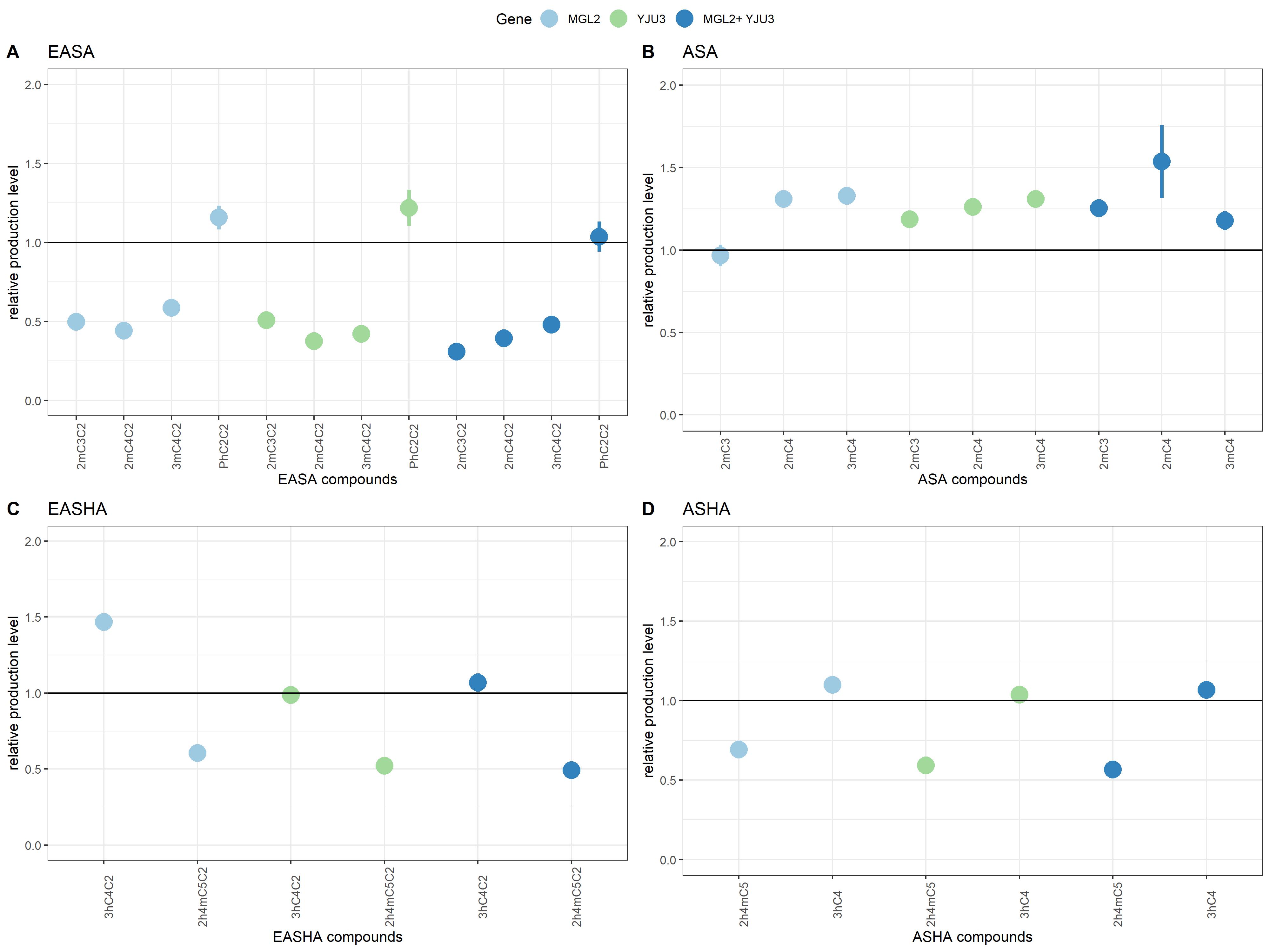

Supplement: Supplementary file 1 [file ijms-22-04026-s001.zip › TABLES et STABLE/Figure S2.jpg]

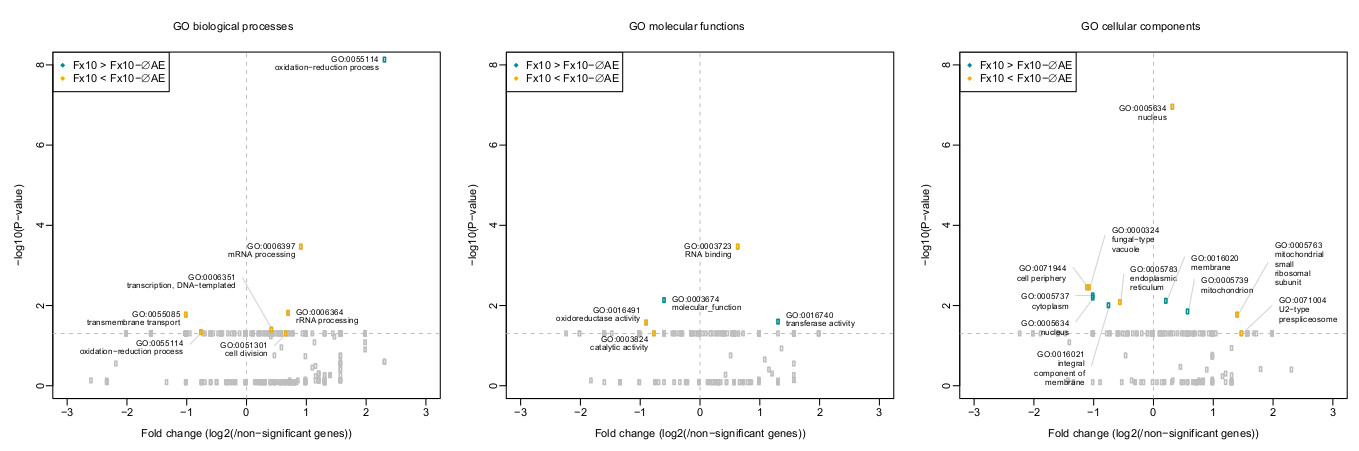

Supplement: Supplementary file 1 [file ijms-22-04026-s001.zip › TABLES et STABLE/Figure S3.jpg]
